# Predictive value of serum HBcrAg in antiviral therapy for chronic hepatitis B

**DOI:** 10.3389/fimmu.2026.1893489

**Published:** 2026-09-03

**Authors:** Shiyu Wang, Hongxiao Hao, Wen Deng, Lu Zhang, Weihua Cao, Ziyu Zhang, Xinxin Li, Yaqin Zhang, Xin Wei, Linmei Yao, Zixuan Gao, Shuojie Wang, Minghui Li

**Affiliations:** 1 Department of Hepatology Division 2, Beijing Ditan Hospital, Capital Medical University, Beijing, China; 2 HBV Infection, Clinical Cure and Immunology Joint Laboratory, Capital Medical University, Beijing, China; 3 Department of Hepatology Division 2, Peking University Ditan Teaching Hospital, Beijing, China

**Keywords:** biological characteristics, chronic hepatitis B, functional cure, HBcrAg, hepatocellular carcinoma (HCC), treatment discontinuation relapse

## Abstract

Chronic hepatitis B (CHB) remains a significant global public health challenge. The goals of its antiviral therapy are gradually shifting from long-term viral suppression toward achieving safer treatment discontinuation, more accurate risk stratification, and deeper functional cure. Traditional serological markers, such as HBV DNA and HBsAg, remain clinically important in CHB. However, their ability to reflect residual viral activity and long-term outcomes is limited after long-term nucleos(t)ide analog (NA) therapy, especially in patients with sustained virological suppression or marked HBsAg decline or clearance.In recent years, hepatitis B core-related antigen (HBcrAg), due to its capacity to reflect transcriptional activity of intrahepatic covalently closed circular DNA(cccDNA) and the state of the residual viral reservoir, has emerged as a notable novel biomarker in the field of CHB. Concurrently, with the development of pegylated interferon (Peg-IFN) add-on strategies and novel combination cure approaches, the potential of HBcrAg in selecting optimal patient populations and monitoring therapeutic efficacy is increasingly recognized. This review focuses on the biological basis of HBcrAg and its main predictive values in CHB antiviral therapy. It also summarizes and prospects its clinical application potential and existing challenges based on current research progress.

## 1 Introduction

Persistent hepatitis B virus (HBV) infection remains one of the major global causes of chronic liver disease, liver cirrhosis, and hepatocellular carcinoma (HCC) (1). Although nucleos(t)ide analogues(NAs) and Peg-IFN have significantly improved treatment outcomes for chronic hepatitis B, current therapies primarily achieve suppression of viral replication. It remains challenging to completely eliminate cccDNA and integrated HBV DNA within hepatocytes, thus falling short of achieving a durable and stable functional cure (2). As treatment goals gradually shift from simply suppressing replication toward safe treatment discontinuation, risk stratification, and cure-oriented management, there is a pressing clinical need for novel biomarkers that can more accurately reflect intrahepatic viral transcriptional activity and the status of the residual viral reservoir. Traditional markers such as HBV DNA and HBsAg hold significant value in disease monitoring but also exhibit certain limitations following long-term antiviral therapy. Especially after prolonged NA-induced suppression, HBV DNA often rapidly declines to undetectable levels, while HBsAg may not adequately reflect the ongoing transcriptional activity of cccDNA (3, 4). In recent years, HBcrAg has gained increasing attention as a novel serological marker in the field of chronic hepatitis B. This is due to its close correlation with intrahepatic cccDNA activity and its ability to provide information on residual viral activity even after HBV DNA seroconversion.

Even when serum HBV DNA is suppressed or becomes undetectable after antiviral therapy, HBcrAg may remain detectable, suggesting the persistence of residual viral transcriptional activity or viral antigen expression. This ongoing viral activity may be associated with chronic hepatic inflammation, hepatocyte injury, and an increased risk of hepatocarcinogenesis (5, 6).

Current studies indicate that (3, 7) the clinical applications of HBcrAg have extended to multiple scenarios, including predicting relapse risk after NAs discontinuation, stratifying HCC risk in patients receiving long-term therapy, assessing Peg-IFN-related responses, and evaluating the depth of functional cure. However, its widespread application still faces challenges such as insufficient standardization of assays, lack of unified optimal cut-off values, and considerable heterogeneity across different study results. This review summarizes the biological basis of serum HBcrAg and its predictive role in antiviral therapy for chronic hepatitis B. By integrating its applications in treatment discontinuation assessment, long-term prognosis evaluation, defining cure depth, and advances in novel anti-HBV therapies, we discuss its clinical translational significance, aiming to provide a basis for precision medicine in chronic hepatitis B.

## 2 Biological characteristics of HBcrAg

### 2.1 Composition and origin

**Figure 1:**
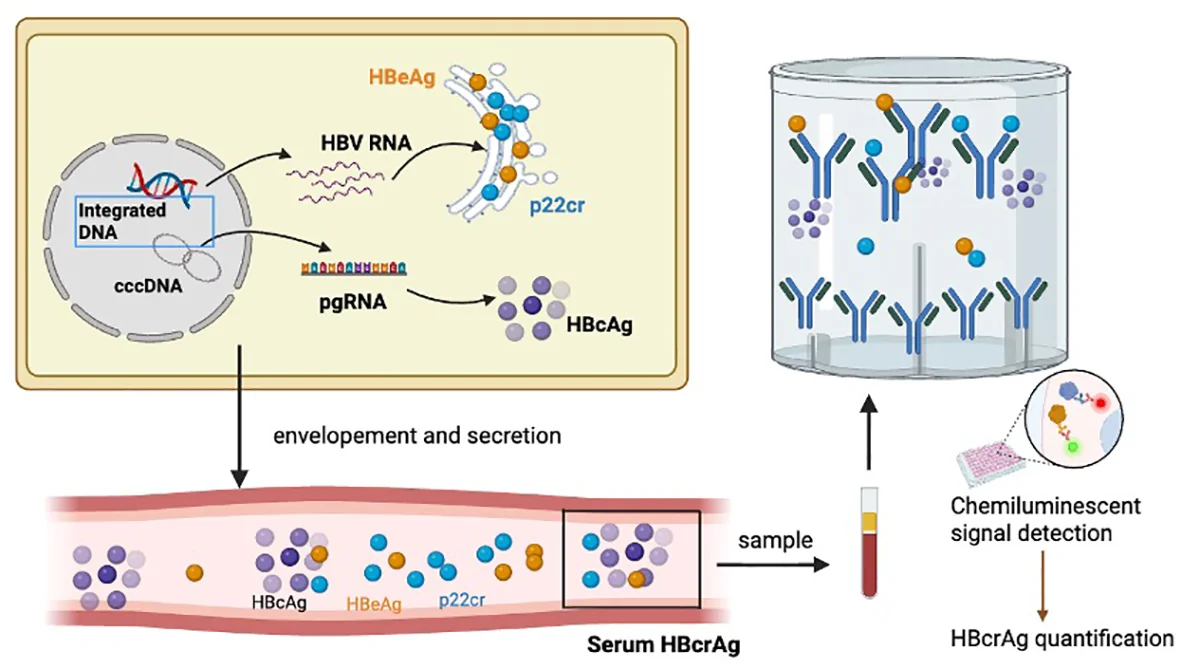
Schematic diagram of the composition, origin, and detection principle of serum HbcrAg. Created with BioRender. cccDNA, covalently closed circular DNA; HBcAg, hepatitis B core antigen; HBeAg, hepatitis B e antigen; HBV, hepatitis B virus; HBcrAg, hepatitis B core-related antigen; pgRNA, pregenomic RNA; p22cr, 22-kDa core-related antigen (precore protein).

Serum HBcrAg is an integrated biomarker composed of three hepatitis B virus core-related proteins: hepatitis B core antigen (HBcAg), hepatitis B e antigen (HBeAg), and the 22 kDa precore protein (p22cr) (8) (**Figure 1**). All three proteins are encoded by the viral precore/core (PreC/C) region and share an identical sequence of 149 amino acid residues. However, they exhibit marked differences in transcriptional and translational start sites, protein processing, and intracellular versus extracellular distribution (9). HBcAg is a structural viral protein involved in nucleocapsid assembly and is primarily located within hepatocytes. HBeAg is a non-structural secretory protein formed after signal peptide cleavage and processing, which can be released into the bloodstream. p22cr is an intermediate product generated during the processing of the precore protein. It lacks the C-terminal arginine-rich domain and is commonly found in empty viral particles that lack HBV DNA (10–12). This combination of molecular origin integration and methodological advantages makes HBcrAg an important serological marker for assessing HBV infection status.

### 2.2 Laboratory setup and assay standardization

Serum HBcrAg is primarily measured using specific chemiluminescent immunoassays (CLIA), particularly the Lumipulse series platforms developed by Fujirebio. These assays use monoclonal antibodies directed against shared core-related epitopes, enabling the simultaneous quantification of HBcAg, HBeAg, and p22cr (13). Therefore, HBcrAg measurement reflects the overall transcriptional and translational activity of the viral precore/core region (14, 15). In clinical practice, HBcrAg testing is usually performed in laboratories equipped with automated chemiluminescent immunoassay systems, using dedicated reagents, calibrators, and quality control materials. Unlike HBV DNA or HBV RNA assays, HBcrAg testing does not require nucleic acid extraction, PCR amplification, or sequencing equipment. However, standardized sample handling, instrument calibration, internal quality control, and trained laboratory personnel are still essential to ensure reliable results.

Currently available assays include the conventional HBcrAg assay (Lumipulse G-HBcrAg) and the recently developed high-sensitivity assay (iTACT-HBcrAg). The iTACT-HBcrAg assay has been reported to be nearly ten times more sensitive than the conventional assay, with the lower limit of detection improved from approximately 2.8 log U/mL to 2.1 log U/mL (3). This increased sensitivity allows HBcrAg to be detected in a higher proportion of HBeAg-negative patients receiving nucleos(t)ide analogue therapy, even when serum HBV DNA is undetectable. Nevertheless, HBcrAg may remain undetectable in some patients even with the iTACT-HBcrAg assay. Differences in analytical sensitivity, calibration, measurement range, and the handling of values below the detection limit may contribute to assay-dependent variability. therefore, cut-off values derived using one assay may not be directly applicable to another. Studies should clearly report the assay platform and its analytical characteristics, including the detection limit and linear range. In addition, the requirement for dedicated instruments, proprietary reagents, maintenance, and trained personnel may increase costs and restrict access, particularly in resource-limited settings. Although centralized testing may provide an interim solution, wider clinical implementation will require assay harmonization, external quality assessment, and formal cost-effectiveness evaluation.

### 2.3 Correlation with intrahepatic viral status

Extensive basic and clinical research consistently indicates that serum HBcrAg levels are closely associated with the state of persistent HBV infection within the liver, particularly the transcriptional activity of cccDNA (16–18). Unlike HBV DNA, which primarily reflects the level of circulating viral replication, HBcrAg, as a composite marker of core/precore region proteins, is more closely associated with the transcriptional activity of cccDNA. Consequently, it is widely regarded as one of the important serological surrogate markers. Multiple studies pairing liver biopsy tissue samples have shown a significant positive correlation between serum HBcrAg levels and intrahepatic cccDNA copy number, total intrahepatic HBV DNA, and pregenomic RNA (pgRNA). In some cohorts, it has been further demonstrated to reflect cccDNA transcriptional activity (16, 17). Among these, Testoni et al. (16) systematically demonstrated, based on paired liver tissue data from CHB patients, the correlation between HBcrAg and cccDNA transcriptional activity, noting that in certain clinical contexts it may outperform indicators like quantitative HBsAg (qHBsAg) in characterizing intrahepatic viral activity.

Notably, the correlation between HBcrAg and residual intrahepatic viral transcriptional activity is particularly prominent in the context of long-term NA therapy (19). NAs potently inhibit reverse transcription, leading to a rapid decrease in serum HBV DNA, but have limited direct effect on eliminating cccDNA. Therefore, even when serum HBV DNA has fallen below the lower limit of detection, HBcrAg can often remain detectable, suggesting it reflects residual transcription or protein expression rather than just the replication process. Honda et al. (20), in a population undergoing NA therapy, indicated that HBcrAg is associated with intrahepatic viral replication and the state of persistent infection, and further observed a link with long-term disease outcomes, thereby reinforcing the clinical significance of HBcrAg in reflecting residual intrahepatic virological activity. Thus, HBcrAg is considered one of the currently available serum surrogate markers that most closely reflects the transcriptional activity of intrahepatic cccDNA, making it particularly suitable for assessing residual virological activity even under profound viral suppression.

Furthermore, some studies and reviews suggest that in HBeAg-negative individuals or during later disease stages, the serum HBcrAg signal might be influenced by factors other than cccDNA, such as transcription associated with viral integration or other protein sources, potentially leading to detectable HBcrAg even when cccDNA levels are low (13, 16, 21). However, evidence regarding the extent of contribution from integrated transcription to HBcrAg levels is not entirely consistent, with more research tending to describe it as a possible contributor with stage-specific and individual heterogeneity. Further studies with paired tissue-serum samples and molecular tracing are needed for clarification.

Overall, existing evidence supports serum HBcrAg acting as a key bridge connecting the intrahepatic virological state with detectable signals in peripheral blood. Its stable correlation with cccDNA load and transcriptional activity provides a crucial basis for its use in evaluating CHB treatment efficacy, guiding treatment discontinuation studies, and exploring endpoints in trials of novel curative strategies.

## 3 The central role of HBcrAg in predicting relapse risk after treatment discontinuation

### 3.1 A potent indicator for predicting relapse after NA discontinuation

For chronic hepatitis B patients receiving long-term NA therapy who have achieved sustained virological suppression, the occurrence of virological relapse (VR) or clinical relapse (CR) after discontinuation depends not merely on whether serum HBV DNA remains long-term undetectable, but more crucially on whether transcriptionally active residual cccDNA persists within the body (1, 22). Unlike HBV DNA, which primarily reflects the outcome of circulating viral replication, HBcrAg is more closely associated with transcriptional activity of intrahepatic cccDNA, thus offering a unique advantage in assessing discontinuation risk. Although earlier studies (7) supported the potential of HBcrAg in predicting treatment response and post-treatment relapse, several limitations continue to constrain its broader clinical application, including heterogeneous outcome definitions across studies, suboptimal performance for predicting HBsAg loss and relapse after NA discontinuation, and the lack of standardized cut-off values. Recent studies have further strengthened its clinical relevance. Current research indicates that a lower HBcrAg level at the end of treatment (EOT) is associated with a greater likelihood of maintaining a sustained response after discontinuation. Conversely, a higher HBcrAg level often suggests a higher probability of virological rebound, biochemical fluctuations, and the need for retreatment following discontinuation (23, 24). The 2025 EASL guidelines have also explicitly stated (1) that when evaluating the feasibility of NA discontinuation, in addition to HBsAg levels, HBcrAg and HBV RNA can further refine risk stratification. This signifies that HBcrAg is progressively transitioning from a research marker to a clinical tool aiding decision-making.

Current studies demonstrate that both baseline and EOT HBcrAg levels are closely associated with post-discontinuation virological relapse (23, 25–27). Prospective studies indicate that the incidence of HBV reactivation is significantly higher in patients who were HBcrAg-positive at baseline compared to those who were HBcrAg-negative, with multivariate analysis confirming that baseline HBcrAg positivity is an independent risk factor for relapse after discontinuation (28).

**Figure 2:**
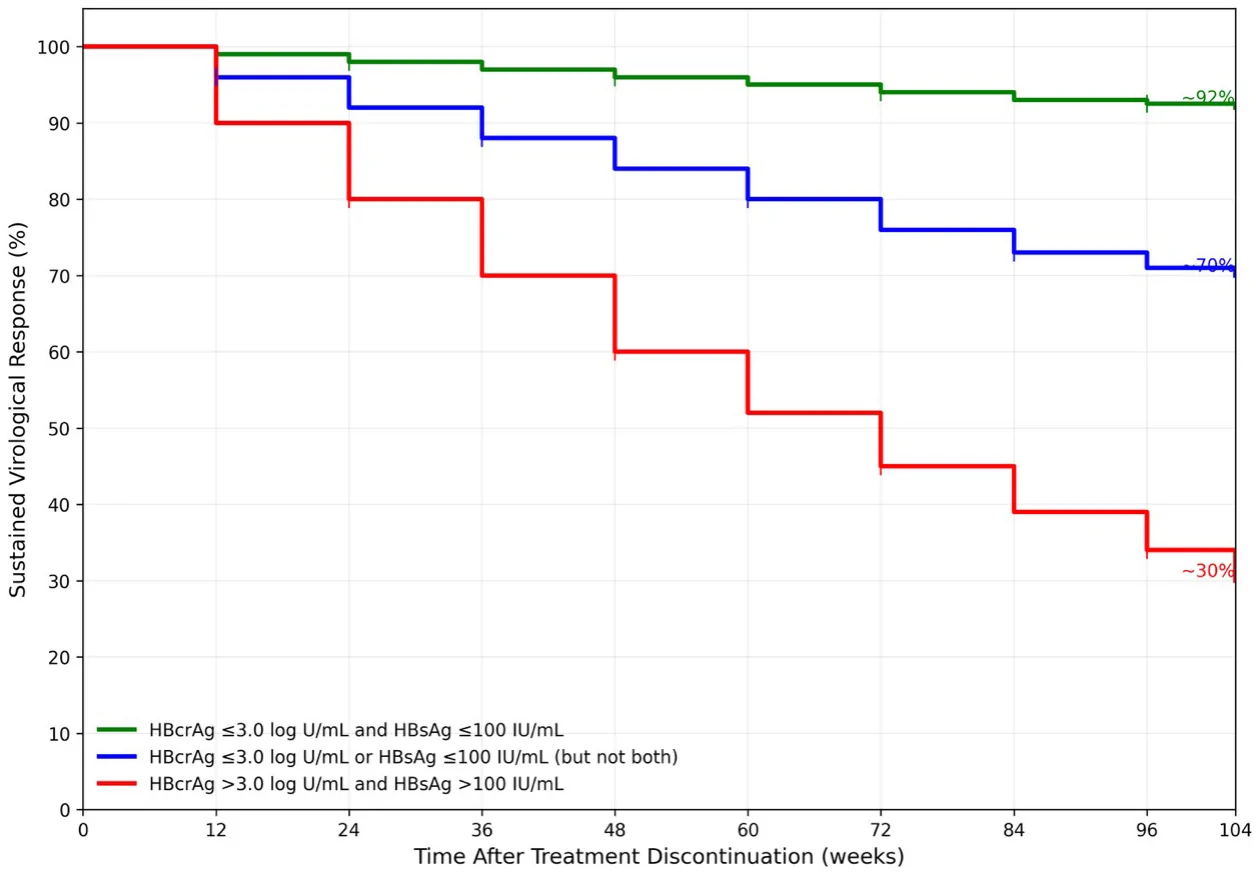
Risk stratification for post-treatment discontinuation relapse based on HBcrAg and HBsAg levels at end of treatment (Kaplan-Meier curve). HBcrAg, hepatitis B core-related antigen; HBsAg, hepatitis B surface antigen.

EOT HBcrAg appears to have particular value in predicting relapse after nucleos(t)ide analog discontinuation. Patients who experience virological or clinical relapse generally have significantly higher EOT HBcrAg levels than those who remain relapse-free, and several studies have identified EOT HBcrAg as an independent predictor of post-treatment relapse (29–31). Although the proposed cut-off values have not yet been fully standardized, EOT HBcrAg levels of approximately 3.7–4.0 log U/mL have been associated with an increased risk of virological relapse, clinical relapse, or hepatitis flare (29–31). Earlier studies also showed that an EOT HBcrAg level below 3.4 log U/mL was associated with a greater likelihood of maintaining off-treatment remission (32). The Japan Society of Hepatology has incorporated HBcrAg into its relapse risk assessment strategy and recommends an EOT level below 3.0 log U/mL as a reference threshold for relatively low relapse risk (32, 33). Accordingly, patients with sustained HBV DNA suppression, low quantitative HBsAg levels, and EOT HBcrAg below 3.0 log U/mL may be more suitable candidates for carefully monitored treatment withdrawal. However, HBcrAg should not be used as a standalone stopping criterion, and decisions regarding NA discontinuation should also consider HBeAg status, treatment duration, fibrosis stage, ALT stability, and the feasibility of close post-treatment monitoring. Combining EOT HBcrAg with quantitative HBsAg may further improve predictive performance (**Figure 2**), supporting its use as an adjunctive marker for relapse risk stratification and the optimization of treatment cessation decisions.

Several cohorts have shown an association between EOT HBcrAg levels and post-treatment relapse. However, most studies were retrospective and conducted mainly in Asian populations, with substantial variation in patient characteristics, treatment regimens, follow-up duration, and relapse definitions. These differences may account for the reported cut-offs of 3.0–4.0 log U/mL and the variable benefit of combining HBcrAg with other biomarkers. Because external validation remains limited, these thresholds should be interpreted within the settings in which they were derived rather than applied as universal criteria for treatment discontinuation.

From a clinical application perspective, HBcrAg is considered a potent predictor for discontinuation not only because it independently indicates relapse risk, but also because it addresses the gap in assessing residual viral activity after HBV DNA becomes undetectable. This enables discontinuation decisions to move beyond reliance solely on traditional virological and biochemical markers. In other words, the true value of HBcrAg lies not merely in predicting whether relapse will occur, but more importantly in helping define which patients are better candidates for entering the treatment discontinuation window, thereby enhancing the safety and precision of finite-duration therapy strategies.

### 3.2 Predictive value in HBeAg-positive patients

In HBeAg-positive chronic hepatitis B, serum HBcrAg levels are associated with viral replication and may help predict HBeAg seroconversion and treatment response (34). A systematic review indicates that the predictive performance of HBcrAg across different clinical endpoints is not entirely consistent; however, its predictive efficacy for HBeAg seroconversion is relatively favorable (7).

Studies suggest that a lower pre-treatment HBcrAg level often indicates a higher likelihood of achieving HBeAg seroconversion during subsequent therapy, while a higher baseline HBcrAg level typically reflects stronger viral replication and a more active transcriptional template pool, suggesting that achieving immune control and seroconversion may require a longer duration of treatment (35) In HBeAg-positive patients receiving NA therapy, a study by Hwang et al. (36) showed that patients who achieved HBeAg seroconversion had significantly lower baseline HBcrAg levels than non-converters. Both baseline HBcrAg and HBcrAg level at 2 years of treatment were identified as independent predictive factors. Their study proposed that a baseline HBcrAg level ≤6.5 log U/mL held certain predictive value for subsequent HBeAg seroconversion.

Beyond the baseline level, the dynamic decline of HBcrAg during treatment may hold greater clinical significance than single measurements. Studies related to Peg-IFN therapy (37, 38)have shown that HBcrAg exhibits a continuous decreasing trend in responders, while the decline is limited in non-responders. Research by Chuaypen et al. (34) on HBeAg-positive patients indicated that monitoring HBcrAg during therapy could help identify individuals with a very low probability of achieving HBeAg clearance and maintaining low HBV DNA levels after Peg-IFN treatment, suggesting HBcrAg’s potential as an early treatment response assessment indicator.

Longitudinal sampling enabled the relationship between changes in HBcrAg and treatment response to be assessed. However, findings have varied across treatment settings. Although several studies identified baseline or on-treatment HBcrAg as an independent predictor of HBeAg seroconversion, its performance across clinical endpoints has been inconsistent and has not consistently exceeded that of qHBsAg or HBV DNA. Interpretation is further limited by differences in treatment regimen, treatment duration, response definition, and the contribution of circulating HBeAg to measured HBcrAg levels.

In HBeAg-positive patients, HBcrAg can serve as an important auxiliary marker for assessing treatment response and predicting HBeAg seroconversion, with its dynamic decline trend holding particularly high clinical value. In practical application, however, it is more suitable to be interpreted in combination with other indicators such as HBsAg and HBV RNA to enhance the predictive accuracy for treatment response and subsequent serological outcomes.

## 4 The unique advantages of HBcrAg in predicting long-term clinical outcomes

### 4.1 Predicting HCC risk: an “integrated” warning signal

Assessment of HCC risk remains an important component of the long-term management of chronic HBV infection. Unlike serum HBV DNA, which reflects circulating viral replication, HBcrAg may remain detectable during sustained virological suppression and provide additional information on residual viral activity and HCC risk (16). Consequently, HBcrAg is not merely a simple virological surrogate marker but rather an “integrative” biomarker that links residual viral transcription, persistent liver injury, and the risk of HCC development.

Current research indicates that the predictive value of HBcrAg for HCC is evident in both untreated patients and those receiving long-term NA therapy (6, 39). Early studies identified an association between elevated HBcrAg levels and an increased risk of subsequent HCC development, and in some scenarios, its predictive capability surpassed or complemented that of HBV DNA (40). Particularly in HBeAg-negative patients in the gray zone, conventional risk stratification based on HBV DNA and ALT has important limitations. These patients generally refer to individuals who are HBeAg-negative but cannot be clearly classified as having either inactive HBV carrier state or HBeAg-negative chronic hepatitis B on the basis of HBV DNA, ALT, and related clinical indicators. Recent studies have proposed HBcrAg-based HCC risk scores that outperform traditional models centered on HBV DNA, suggesting that HBcrAg may better identify patients with apparently low circulating viral burden but persistent intrahepatic viral transcriptional activity (6).

The advantage of HBcrAg is more pronounced in patients undergoing long-term antiviral therapy. Research by Hosaka et al. (39) showed that among patients receiving long-term NA therapy with sustained virological suppression, those whose HBcrAg levels remained persistently high during treatment had a significantly increased risk of developing HCC. This indicates that suppression of HBV DNA does not equate to the simultaneous elimination of carcinogenic risk. Subsequent multiple studies further confirmed (41, 42) that in populations receiving standard NA therapy, both pre-treatment and on-treatment (e.g., at 1 year) HBcrAg levels can provide additional information for HCC risk stratification. This utility is particularly clinically meaningful in non-cirrhotic patients or populations where traditional risk stratification is not clearly defined.

It is important to note that HBcrAg should not be interpreted as a single indicator capable of replacing all traditional HCC risk factors. Current evidence more strongly supports its integration into a multidimensional risk assessment framework, to be used in conjunction with factors such as age, sex, cirrhosis status, family history, alcohol consumption, and other virological markers (43–45). A systematic review has indicated that HBcrAg positivity or higher levels are associated with an increased risk of HCC development, with hazard ratios exceeding 3-fold in some studies (39). However, the optimal cut-off values vary across different studies, suggesting that its clinical application still requires interpretation based on the specific population context, HBeAg status, and treatment status.

Several longitudinal studies with extended follow-up have linked higher HBcrAg levels to HCC risk after adjustment for established risk factors. However, most were retrospective, and residual confounding cannot be excluded. Findings have varied between untreated and NA-treated populations, and the added value of HBcrAg over established HCC risk scores remains uncertain. This heterogeneity may reflect differences in HBV genotype, ethnicity, cirrhosis prevalence, timing of measurement, and selected cut-offs. HBcrAg should therefore be considered an additional risk marker rather than a basis for changing HCC surveillance strategies.

Therefore, for patients receiving long-term NA therapy with undetectable HBV DNA, regular monitoring of HBcrAg aids in HCC risk stratification. Patients with high HBcrAg levels, even those with normal liver function and undetectable viral load, should be considered at high risk for HCC. This warrants intensified surveillance, such as shortening the ultrasound examination interval to every 4–6 months, and consideration of more proactive secondary prevention strategies.

### 4.2 Assessing long-term prognosis after functional cure

HBcrAg may also provide prognostic information in patients who have achieved functional cure. HBsAg clearance is an important treatment milestone, but it does not necessarily indicate complete elimination of HBV genetic material. In some patients, low-level HBcrAg can still be detected after HBsAg clearance, suggesting residual viral activity. These patients may continue to carry a higher long-term risk of HCC than those in whom HBcrAg is completely undetectable (46–48). Suzuki et al. (46) reported that ultra-sensitive HBcrAg may remain detectable in some patients even after HBsAg clearance. This residual signal is thought to reflect ongoing viral biological activity and may be linked to later HCC development. Thus, HBcrAg may help distinguish patients with HBsAg seroclearance who still carry residual risk from those who have achieved a deeper virological remission.

HBcrAg may also provide information on the durability of functional cure. In patients who achieved HBsAg clearance after Peg-IFN-based therapy, Huang et al. (49) reported that lower end-of-treatment HBcrAg levels combined with higher anti-HBs titers were associated with sustained response and durable functional cure. This finding suggests that reduced residual intrahepatic viral activity, together with stronger humoral immune control, may contribute to the long-term stability of functional cure.Therefore, among patients receiving pegylated interferon-based therapy, those with low HBcrAg levels and high anti-HBs titers at the end of treatment are more likely to achieve sustained clinical cure. However, specific HBcrAg thresholds for pretreatment patient selection have not yet been standardized.

These findings suggest that HBcrAg reflects residual viral burden and may provide useful information on the long-term stability of functional cure.

HBcrAg may also help identify patients with a potentially unstable functional cure after NA discontinuation. In a recent study of non-cirrhotic patients who discontinued NAs after achieving HBsAg clearance, most patients remained HBsAg-negative during follow-up (50). However, HBcrAg was detectable at treatment withdrawal in all patients who subsequently experienced HBsAg seroreversion. This finding suggests that detectable HBcrAg may reflect persistent viral transcriptional activity or a residual viral reservoir despite apparent functional cure. Patients with detectable HBcrAg in this setting may therefore require closer post-withdrawal serological monitoring than those with undetectable HBcrAg.

Therefore, from a long-term management perspective, the unique advantage of HBcrAg lies in its ability to extend the assessment of whether a cure is achieved further to evaluating whether the cure is sufficiently deep, sufficiently stable, and whether it still carries a residual risk of long-term adverse outcomes. Compared to defining the endpoint solely by HBsAg seroclearance, incorporating HBcrAg can elevate the connotation of a functional cure from a static serological outcome to a dynamic, long-term risk stratification. This characteristic gives HBcrAg significant potential in post-cure follow-up, identification of relapse risk, and optimization of HCC surveillance strategies.

A more rational approach for future application may involve integrating HBcrAg into a comprehensive predictive model alongside HBsAg, HBsAb, HBV RNA, and clinical risk factors. This would enable a more precise assessment of the long-term prognosis after cure.

Based on these biological and methodological considerations, HBcrAg has been evaluated across multiple clinical scenarios, including treatment discontinuation, treatment response prediction, HCC risk stratification, and functional cure assessment. The key studies are summarized in **Table 1**.

**Table 1:** Summary of key studies evaluating the clinical utility of HBcrAg in chronic hepatitis B.

Study	Population characteristics	Treatment setting	Endpoint	Main findings related to HBcrAg
Testoni et al. (16)	CHB patients with paired serum and liver tissue samples	Mixed clinical settings	Intrahepatic cccDNA and cccDNA transcriptional activity	Serum HBcrAg correlated with intrahepatic cccDNA transcriptional activity, supporting its role as a non-invasive surrogate marker of residual intrahepatic viral activity.
Chen et al. (17)	CHB patients with liver biopsy samples	Untreated or treated CHB	Intrahepatic cccDNA	Serum HBcrAg showed a positive correlation with intrahepatic cccDNA, indicating its potential value in reflecting the intrahepatic viral reservoir.
Honda et al. (20)	CHB patients receiving long-term NA therapy	NA therapy	Intrahepatic viral replication and HCC development	HBcrAg remained informative despite HBV DNA suppression and was associated with residual intrahepatic HBV replication and subsequent HCC development.
Hsu et al. (30)	HBeAg-negative CHB patients who discontinued entecavir or tenofovir after sustained viral suppression	NA discontinuation	Off-treatment relapse	Combining end-of-treatment HBcrAg with HBsAg improved prediction of relapse after NA withdrawal; low EOT HBcrAg was associated with a lower relapse risk.
Brakenhoff et al. (24)	CHB patients who discontinued NA therapy	NA discontinuation	Off-treatment ALT flares	EOT HBsAg, HBcrAg and HBV RNA were associated with the risk of ALT flares after treatment withdrawal, suggesting that HBcrAg may complement other viral biomarkers.
Hume et al. (23)	CHB patients stopping NA therapy	NA discontinuation	Hepatitis flare after treatment cessation	Higher EOT HBcrAg levels predicted hepatitis flare after stopping NA therapy, supporting its use in pre-withdrawal risk assessment.
Hwang et al. (36)	HBeAg-positive CHB patients receiving antiviral therapy	NA-based antiviral therapy	HBeAg seroconversion	Lower baseline HBcrAg and lower on-treatment HBcrAg were associated with subsequent HBeAg seroconversion; baseline HBcrAg ≤6.5 log U/mL was proposed as a useful predictive threshold.
Chuaypen et al. (34)	HBeAg-positive CHB patients	Peg-IFN therapy	HBeAg clearance and treatment response	On-treatment HBcrAg monitoring helped identify patients unlikely to achieve HBeAg clearance or favorable virological response after Peg-IFN therapy.
Ma et al. (38)	HBeAg-positive CHB patients	Peg-IFNα-2b therapy	HBeAg seroconversion	HBcrAg helped identify patients who were unlikely to achieve HBeAg seroconversion during Peg-IFNα-2b therapy.
Tada et al. (6)	CHB patients under long-term follow-up	Untreated or mixed treatment background	HCC development	Higher HBcrAg levels predicted future HCC development and provided additional prognostic information beyond conventional viral markers.
Hosaka et al. (39)	CHB patients receiving long-term NA therapy	NA therapy	HCC incidence	Persistent or elevated HBcrAg during NA therapy was associated with increased HCC risk, even in patients with sustained virological suppression.
Tseng et al. (40)	HBeAg-negative gray-zone CHB patients	Untreated or clinically indeterminate patients	HCC risk prediction	An HBcrAg-based risk score performed better than HBV DNA-based models for predicting HCC in HBeAg-negative gray-zone patients.
Suzuki et al. (46)	Patients with HBsAg seroclearance	Post-HBsAg seroclearance follow-up	HCC development after HBsAg loss	Detectable ultra-sensitive HBcrAg after HBsAg seroclearance suggested residual viral biological activity and was associated with subsequent HCC development.
Huang et al. (49)	CHB patients who achieved HBsAg clearance after Peg-IFN-based therapy	Peg-IFN-based therapy	Durable functional cure	Lower EOT HBcrAg combined with higher anti-HBs levels was associated with durable functional cure, suggesting that HBcrAg may help assess cure depth and stability.

CHB, chronic hepatitis B; HBcrAg, hepatitis B core-related antigen; HBV, hepatitis B virus; NA, nucleos(t)ide analogue; Peg-IFN, pegylated interferon; HBeAg, hepatitis B e antigen; HBsAg, hepatitis B surface antigen; anti-HBs, hepatitis B surface antibody; cccDNA, covalently closed circular DNA; HCC, hepatocellular carcinoma; EOT, end of treatment; ALT, alanine aminotransferase.

## 5 Future research directions and challenges

### 5.1 Assay standardization and threshold establishment

Although HBcrAg has demonstrated significant predictive value in the antiviral treatment of chronic hepatitis B, its broader integration into routine decision-making is primarily hindered by the lack of full standardization for both the assay platforms and the established thresholds. The Lumipulse series assays are currently the most commonly used in clinical practice and research. Recently, the iTACT-HBcrAg has emerged, lowering the limit of detection from approximately 2.8 log U/mL for conventional HBcrAg assay(G-HBcrAg) to 2.1 log U/mL, thereby enabling the identification of residual viral activity at even lower levels (3, 51, 52). However, the sensitivity, linear range, and interpretation of low-value intervals are not entirely equivalent across different platforms. This means that the definitions of positivity, low-level detectability, and optimal cut-offs reported in various studies cannot be directly extrapolated or compared simply.

This issue is particularly pronounced when applying specific thresholds. Existing studies have proposed multiple HBcrAg cut-off points for different clinical endpoints, such as risk stratification for treatment discontinuation relapse, HCC risk stratification, and long-term risk assessment after functional cure. These commonly cited cut-offs range from approximately 2.7 to 4.9 log U/mL. However, these thresholds are often influenced by factors including patient population characteristics, treatment type, timing of follow-up, and differences in detection methodologies (3, 53). The 2025 EASL guidelines (1) explicitly state that while increasing evidence supports the use of HBcrAg for risk stratification in candidates for NA discontinuation, definitive cut-offs for clinical decision-making have not yet been established. Furthermore, cohort heterogeneity and differences in assay characteristics can both affect the interpretation of results.

Before HBcrAg can be widely adopted in clinical practice, several methodological and interpretive issues remain to be resolved. Assay calibration should be improved to allow better comparability across platforms, and clinically relevant thresholds need to be defined according to HBeAg status, treatment modality, and intended endpoint. In addition, HBcrAg is unlikely to be best interpreted as a single absolute value. A more informative approach may be to consider baseline levels, the magnitude of on-treatment decline, and end-of-treatment values in combination. These refinements will be important for moving HBcrAg from a research biomarker toward a standardized tool for individualized management of chronic hepatitis B.

### 5.2 Synergistic comparison with other novel biomarkers

From a biological perspective, the core advantage of HBcrAg lies in its closer correlation with the transcriptional activity of intrahepatic cccDNA. In contrast, a portion of HBsAg can originate from integrated HBV DNA. consequently, in the later stages of the disease, particularly in HBeAg-negative patients and after long-term NA therapy, HBsAg does not always adequately reflect the activity of the residual replicative template (54, 55). Recent reviews note that the correlation of HBcrAg with intrahepatic cccDNA is generally stronger than that of HBsAg and HBV DNA. Even when HBV DNA becomes undetectable after long-term NA suppression, HBcrAg often remains detectable, thereby providing additional information for identifying patients who appear virologically quiescent but still harbor active viral transcription (27, 56, 57).

However, these findings do not indicate that HBcrAg is superior to other available biomarkers in every clinical setting. To better define its clinical role, we compared HBcrAg with the major predictive biomarkers currently used in chronic hepatitis B (**Table 2**). As shown in **Table 2**, the main strength of HBcrAg lies in its complementary rather than substitutive value, particularly in providing additional insight into residual viral transcriptional activity in patients receiving long-term antiviral therapy or being considered for treatment discontinuation. Among the available biomarkers, HBV RNA is currently the closest comparator and complement to HBcrAg. Studies indicate that in NA discontinuation studies, both HBcrAg and HBV RNA can help predict partial cure and relapse risk after treatment cessation (58, 59). In some cohorts, when used in combination with qHBsAg, their ability for risk stratification surpasses that of any single marker (60). The EASL 2025 guidelines (1) conclude that lower or undetectable HBcrAg, particularly when combined with low HBsAg levels, is associated with a lower relapse risk. Some treatment discontinuation cohort studies suggest that HBV RNA combined with qHBsAg demonstrates good risk stratification ability for certain endpoints, whereas, by comparison, the combination of HBcrAg and qHBsAg has not shown consistently superior performance across all studies (61, 62). In other words, the practical positioning of HBcrAg is more likely to be a key component within a combined biomarker system, rather than a standalone replacement for other indicators.

**Table 2:** Comparison of HBcrAg with currently available predictive biomarkers in chronic hepatitis B.

Biomarker	primarily reflects	Clinical utility	Main strengths	Main limitations
HBcrAg	Composite precore/core-related antigen signal reflecting residual viral transcriptional activity and cccDNA-related activity	HCC risk stratification, NA cessation assessment, treatment response, depth of functional cure	Retains clinical information when HBV DNA is suppressed; correlates with cccDNA-related activity; useful in long-term treated patients	Cut-offs are not yet fully standardized; assay/platform heterogeneity exists; performance for predicting HBsAg loss alone is modest
HBV DNA	Circulating viral replication	Treatment initiation, virological response, relapse monitoring, disease phase classification	Widely standardized; central to routine management	Often becomes undetectable during NA therapy and may underestimate residual transcriptional activity and persistent oncogenic risk
ALT	Hepatocellular injury/necroinflammation	Treatment decisions, clinical relapse, biochemical monitoring	Inexpensive and universally available	Non-specific; influenced by metabolic disease, alcohol, drugs, and coexisting liver conditions; does not directly reflect viral activity
HBeAg	Active precore/core expression and immune-active phase	Disease phase classification, prediction of HBeAg seroconversion	Familiar and easy to interpret in HBeAg-positive disease	Limited utility in HBeAg-negative patients and low-replication states
qHBsAg	Total HBsAg burden from both cccDNA and integrated HBV DNA	Functional cure assessment, NA cessation selection, selected risk stratification settings	Particularly useful for predicting HBsAg loss and defining low-HBsAg states	Does not specifically reflect cccDNA transcriptional activity because integrated HBV DNA contributes to HBsAg production
Serum HBV RNA	cccDNA transcriptional activity and replicative intermediates	NA cessation risk stratification, treatment response, residual viral activity assessment	Biologically close to cccDNA transcription; useful in off-treatment risk assessment	Assays are less standardized and less widely available than HBV DNA; real-world access remains limited
Intrahepatic cccDNA	Viral reservoir/persistence	Research reference standard	Most directly linked to viral persistence	Requires liver biopsy; invasive; not practical for routine follow-up; laboratory methods vary

HBcrAg, hepatitis B core-related antigen; HBV, hepatitis B virus; HCC, hepatocellular carcinoma; NA, nucleos(t)ide analogue; ALT, alanine aminotransferase; HBeAg, hepatitis B e antigen; qHBsAg, quantitative hepatitis B surface antigen; cccDNA, covalently closed circular DNA. This table provides a practical comparison of the currently available predictive biomarkers in chronic hepatitis B based on current evidence.

Beyond HBV RNA and qHBsAg, anti-HBc, anti-HBs, and immunological markers are also rapidly entering this field. The EASL 2025 guidelines have listed anti-HBc, inflammatory markers, and immune markers as exploratory directions for predicting post-discontinuation response, while also emphasizing that these markers currently lack sufficient standardization (**Table 2**). It is noteworthy that Peg-IFN-related studies also suggest that a lower baseline HBcrAg is associated not only with more pronounced HBsAg decline but also with better restoration of HBV-specific T-cell function (49, 63). This indicates that HBcrAg may possess the dual attributes of both a virological load indicator and a predictor of response to immunotherapy.

A more clinically meaningful direction for the future may not be simply comparing the superiority of various biomarkers, but rather integrating virological, immunological, and clinical characteristics to construct more comprehensive and reliable predictive models.

### 5.3 Role in novel treatment strategies

As the treatment goal for chronic hepatitis B shifts from long-term viral suppression to the pursuit of a functional cure, the role of HBcrAg is also evolving from a traditional disease monitoring marker to a companion biomarker in new drug development. Current novel therapeutic strategies primarily revolve around two main directions: firstly, virus-targeting agents that further reduce antigen load, such as siRNA, antisense oligonucleotides, and capsid assembly modulators; and secondly, immunomodulatory agents that restore the host antiviral response. Expert consensus suggests (2) that the key to achieving a functional cure lies in simultaneously reducing viral protein load and restoring host immune control. Given that HBcrAg better reflects the transcriptional activity of cccDNA, it holds promise as a non-invasive indicator for assessing whether such combination therapies genuinely impact the activity of the viral reservoir.

Existing clinical studies provide initial support for this view. In individuals already achieving virological suppression, a lower baseline HBcrAg is associated with a more significant decline in HBsAg and better restoration of HBV-specific T-cell function following add-on Peg-IFN therapy, suggesting HBcrAg could be used to identify patients more likely to benefit from immunomodulatory treatments (63). Concurrently, the efficacy of novel combination regimens is improving. In a Phase II study of elebsiran combined with PEG-IFNα, the HBsAg loss rate during follow-up after 24 weeks of treatment was significantly higher than with PEG-IFN monotherapy (64). Furthermore, studies on bepirovirsen sequential therapy with PEG-IFN suggest that this sequential combination approach may reduce the risk of relapse after stopping RNA-targeted therapy alone (65).

Although these trials currently primarily use HBsAg and HBV DNA as key endpoints, based on the mechanisms and application logic, HBcrAg is well-suited for integration into the stratification criteria, early response assessment, and post-treatment relapse monitoring systems of such studies. However, it should also be recognized that the role of HBcrAg in novel therapies is still in a phase of clear potential but accumulating evidence. Firstly, many new drug studies still primarily use HBsAg decline or clearance as the main efficacy endpoint, with HBcrAg often serving as a secondary exploratory measure. Secondly, the magnitude and kinetics of the effect on HBcrAg may differ across drugs with varying mechanisms, and a unified definition of an HBcrAg response pattern is still lacking. Thirdly, novel therapies often involve drug combinations; interpreting whether changes in HBcrAg represent suppression of cccDNA transcription, reduction in antigen load, or an indirect effect of enhanced immune control still requires correlation with HBV RNA, qHBsAg, and even intrahepatic markers.

In other words, HBcrAg is highly likely to become an important nodal biomarker in the era of functional cure, but its optimal use still requires further validation embedded within prospective clinical trials.

## 6 Conclusions and perspectives

Serum HBcrAg is an important novel biomarker in antiviral therapy for chronic hepatitis B, possessing both biological rationale and clinical predictive value. Compared to traditional HBV DNA testing, HBcrAg can further reflect transcriptional activity of cccDNA and the state of the residual viral reservoir, even in the context of virological suppression or significant HBsAg decline, thus offering unique advantages in stages where traditional indicators are insufficient for comprehensive assessment. HBcrAg holds significant potential for application in various areas, including treatment discontinuation decision-making, HCC risk stratification, and the evaluation of cure depth.

Nevertheless, current evidence still favors HBcrAg as a complementary rather than a standalone biomarker, with greater utility when interpreted together with qHBsAg, HBV RNA, and conventional clinical parameters. Further standardization of assays, validation of clinically meaningful thresholds, and prospective studies across diverse populations will be essential to define its role more clearly in routine practice.

Most clinical studies on HBcrAg have been conducted in adult patients with CHB, with early evidence largely derived from Asian cohorts (66). In recent years, the scope of research has gradually expanded to populations in Europe, North America, and Africa (67). However, the optimal interpretive thresholds may not be fully consistent across different geographic regions and patient populations. HBV genotype may partly contribute to this variability. In a large European multicenter cohort of HBeAg-negative individuals, HBcrAg levels differed across genotypes. however, genotype-specific cut-offs did not significantly improve diagnostic accuracy compared with the overall threshold (68). Moreover, no significant difference in HBcrAg levels was observed between genotypes B and C in a Chinese cohort (69). These findings suggest that the independent effect of HBV genotype on HBcrAg expression and predictive performance remains uncertain and may be smaller than the effects of HBeAg status, disease phase, and viral load. A study (70) involving cohorts from the United Kingdom and South Africa showed that the diagnostic performance of the same HBcrAg threshold varied between populations. Nevertheless, these cohorts differed not only in ethnicity and HBV genotype distribution but also in HBeAg status, HIV coinfection, and viral load. therefore, the observed differences cannot be attributed to genotype or ethnicity alone. Population-specific validation may consequently be required before applying a uniform HBcrAg threshold across different clinical settings. In addition, studies in special populations, particularly pregnancy-related cohorts, have recently emerged. These studies suggest that dynamic changes in HBcrAg and pgRNA levels in pregnant women with chronic hepatitis B may reflect different disease phases and virological characteristics during pregnancy and the peripartum period. This indicates a potential role for HBcrAg in pregnancy monitoring and postpartum management. However, evidence in these populations remains limited, and validated HBcrAg thresholds for predicting maternal outcomes, postpartum hepatitis flares, or mother-to-child transmission are not yet available. Prospective multicenter studies including patients with diverse demographic, geographic, and virological characteristics are needed to establish appropriate thresholds and define the clinical value of HBcrAg in these settings.

In clinical practice, the value of HBcrAg is reflected in multiple aspects. Among patients receiving long-term NA therapy, particularly those who are HBeAg-negative with stable virological suppression, lower HBcrAg levels at the end of treatment, especially when accompanied by low HBsAg levels, can help identify individuals likely to maintain a sustained response and exhibit a reduced risk of relapse after therapy discontinuation. Persistent elevation of HBcrAg at baseline or during treatment also signals a higher risk of hepatocellular carcinoma and provides important supplementary information to traditional risk assessment systems. Furthermore, HBcrAg offers a valuable metric in evaluating functional cure, as sustained undetectability or very low levels may more accurately reflect the depth of virological control than HBsAg clearance alone.

Of course, the widespread clinical application of HBcrAg still faces several challenges, including the lack of fully standardized assay platforms, inconsistent optimal cut-off values across different studies, and some heterogeneity in predictive performance among different populations and under various treatment contexts. Therefore, future efforts must rely on large-scale, multi-center, prospective studies to further clarify its standardized testing procedures and stratified cut-off points, and to promote its integrated use with qHBsAg, HBV RNA, and immunological markers.

**Figure 3:**
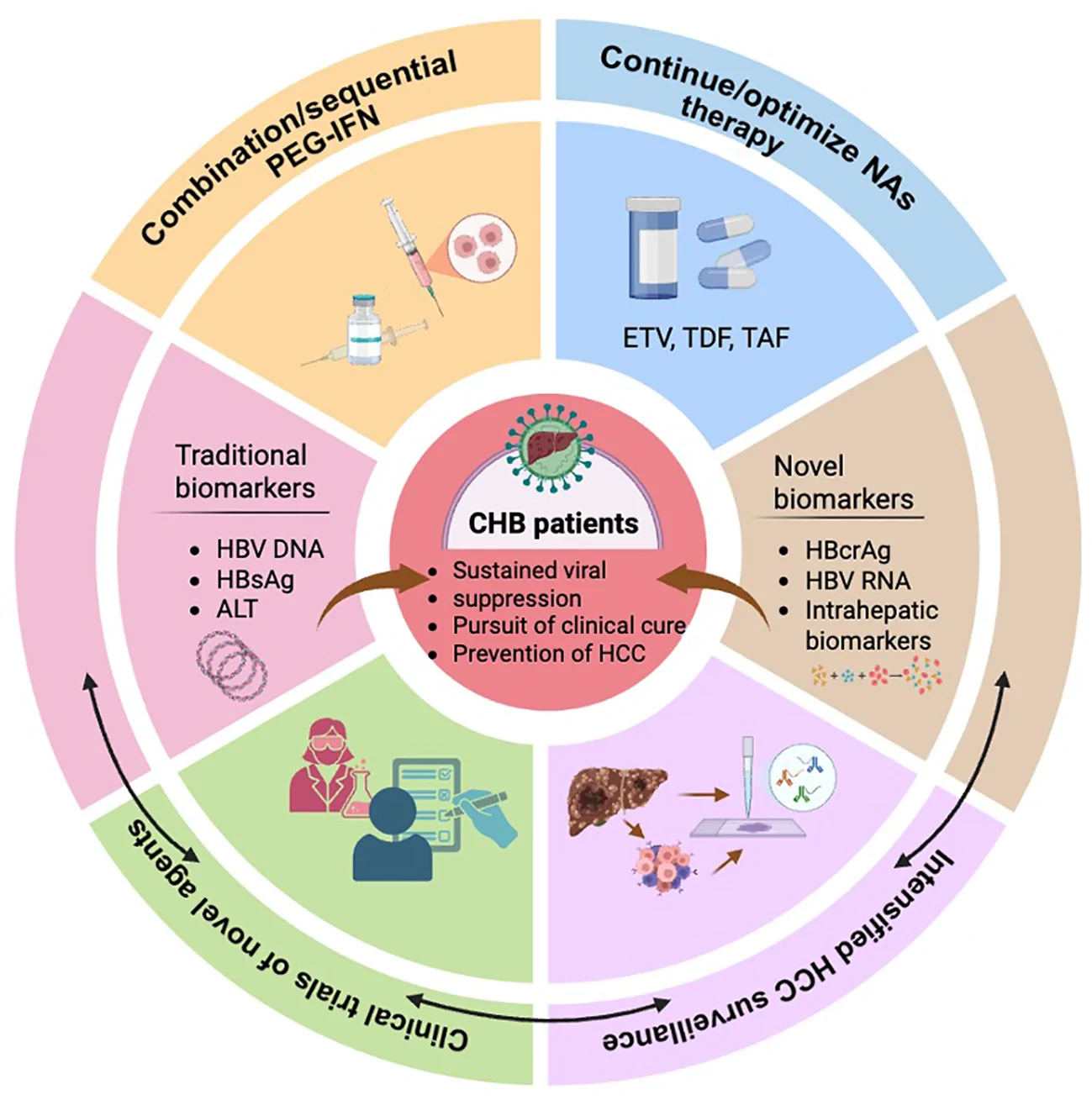
Future vision for individualized and precision management of chronic hepatitis B based on multi-dimensional biomarkers. Created with BioRender. ALT, alanine aminotransferase; CHB, chronic hepatitis B; ETV, entecavir; HBsAg, hepatitis B surface antigen; HBcrAg, hepatitis B core-related antigen; HBV, hepatitis B virus; HCC, hepatocellular carcinoma; NA, nucleos(t)ide analogue; Peg-IFN, pegylated interferon; TAF, tenofovir alafenamide; TDF, tenofovir disoproxil fumarate.

Overall, HBcrAg is expected to evolve gradually from a promising surrogate marker into an important tool for the precise stratification and individualized management of chronic hepatitis B. With advancements in highly sensitive detection technologies and the development of novel anti-HBV treatment strategies, the role of HBcrAg in optimizing treatment cessation strategies, enhancing cancer risk, and defining higher-level cure endpoints is highly anticipated. By integrating multi-dimensional information, we will undoubtedly be able to construct a unique ‘virus-host interaction map’ for each CHB patient, thereby ushering in a new era of truly individualized and precision management for chronic hepatitis B (**Figure 3**).
